# Evaluation of a Three-dimensional printable and two CAD/CAM hybrid resin materials used as permanent single tooth restoration: An in-Vitro study

**DOI:** 10.1186/s12903-025-06544-8

**Published:** 2025-07-21

**Authors:** Noha Saadoun, Mohamed Sherif Mohamed Salah Farag, Rania El-Saady Badawy

**Affiliations:** 1 Department of Dental Biomaterials, Faculty of Dentistry, Suez University, P.O.Box: 43221, Suez, Egypt; 2 Department of Pediatric and Preventive Dentistry and Dental Public Health, Faculty of Dentistry, Suez Canal University, Ismailia, Egypt; 3 Department of Dental Biomaterials, Faculty of Dentistry, Suez Canal University, Ismailia, Egypt

**Keywords:** Degree of conversion, Flexural strength, Fractography, 3D printing, Dental CAD/CAM materials

## Abstract

**Objectives:**

To evaluate and compare three commercially available hybrid resin materials used for additive and subtractive production (CAD/CAM applications) of permanent single-tooth restorations.

**Materials and methods:**

One-hundred and twenty-five bar-shaped specimens (14 mm length, 2 mm width, and 2 mm thickness) of 3 different hybrid resin materials were used in this study, being classified into 5 groups (n = 25). Group I: Lava Ultimate, group II: Vita Enamic, group III: Flexcera Smile Ultra^+^ A, group IV: Flexcera Smile Ultra^+^ B and group V: Flexcera Smile Ultra^+^ C. Lava Ultimate and Vita Enamic specimens were prepared with a low-speed water-cooled diamond microsaw. A digital light processing 3D printer was utilized to print Flexcera Smile Ultra^+^ specimens of 3 different printing angles (0, 45, 90 angles). Specimens were investigated regarding degree of conversion (DC), surface roughness, flexural strength (FS) & modulus (FM) and fractographic analysis. The collected data was tabulated and subjected to statistical analysis. One Way ANOVA test was used to analyze data statistically.

**Results:**

Flexcera Smile Ultra^+^ B group showed the highest statistically DC value (46.40). Vita Enamic group showed the highest statistically surface roughness value (0.360). Lava Ultimate group showed the highest statistically FS value (169.87), however Vita Enamic group showed the highest statistically FM value (13.45). Scanning electron microscope images of fractured specimens revealed a compression curl in the upper part and the fracture origin in the lower portion of the surface, which denotes specimens bending. Microcracks voids, arrest lines and hackle lines are also detected. Fractured specimens showed different crack propagation behaviors.

**Conclusions:**

Different printing angles (orientations) strongly affected degree of conversion, flexural strength and modulus of the 3D printed material. FS and FM of the 3D printed material were lower than those of CAD/CAM blocks. The different groups showed variable degrees of surface roughness. The fracture surfaces of the different groups revealed common features, indicating bending and showing different crack pathways.

## Introduction

Human teeth are essential for chewing, occlusion, and appearance. Frequently, indirect restorations are needed to restore tooth appearance and functionality, especially in cases of significant tooth decay due to caries, endodontic treatment, tooth attrition and abrasion, or even coronal fractures. With the constantly evolving technology, a broad application of computer-aided design and computer-aided manufacturing (CAD/CAM) machineries to fabricate indirect single tooth restorations in both laboratories and dental offices has been noticed [1].

Utilizing computer numerical control machines, subtractive manufacturing of solid substances (blocks and discs) is a predominant production technique [2]. Metals, ceramics, and resin composites are among the materials used for the subtractive production of permanent restorations [3–5]. Subtractive CAD/CAM technologies have led to major improvements in dentistry [2]. They enabled the production of reliable restorations with accurate dimensions [6], together with a concomitant reduction in the required manufacturing time [2].

Still, the subtractive method has some drawbacks, as the motion range of the cutting device and the size of the cutting bur limit the millable shape [2, 7]. This causes waste of raw material, and the loss of unused portions of blocks, added to a difficulty in recycling excess material. Furthermore, the milling process itself, may cause heavy wear of cutting tools, and possible resultant microscopic cracks appearing in objects, associated with weakened restoration [8].

Nowadays, additive manufacturing (AM) is used as an alternative approach to the subtractive manufacturing technique. It is also called three-dimensional (3D) Printing or Rapid Prototyping, which experienced a revolutionary expansion in dentistry with the aid of CAD/CAM technology [9]. By using this technique, metallic frameworks, models, provisional restorations, splints, removable and fixed prosthetics are frequently manufactured [2, 3].

Among the different AM techniques, digital light processing (DLP) and stereolithography (SLA) are the most promising in terms of the production of accurate designs with fine surface finishing [10]. The three-dimensional additive printing method produces 3D objects by layering cross-sectional slices to form the definitive object [11], which facilitates the production of objects with complex structures [12, 13].

Even though that AM has many advantages such as material saving and independence of the milling equipment, it still has some disadvantages that are rarely discussed in dentistry. Due to the layered fabrication technique applied, mechanical anisotropy and low filler content are the main drawbacks of AM [14, 15].

Different parameters, such as printing orientation, layer thickness and post curing can affect the final printing results. The setting of the printing orientation strongly affects the properties of the material, product accuracy and biocompatibility [16–18]. According to many studies, a printed layer direction perpendicular to the load direction showed better compressive strength results of the material compared to being parallel [11, 19]. A new 3D printable light-cured hybrid nanoceramic resin material was introduced in the dental market. Up till now, limited researches had been conducted on it.

The null hypothesis was that there would be no significant differences in degree of conversion, surface roughness, flexural strength & modulus and fractographic examination between hybrid resin materials for printing and for milling.

## Materials and methods

### Materials and devices

**Fig. 1:**
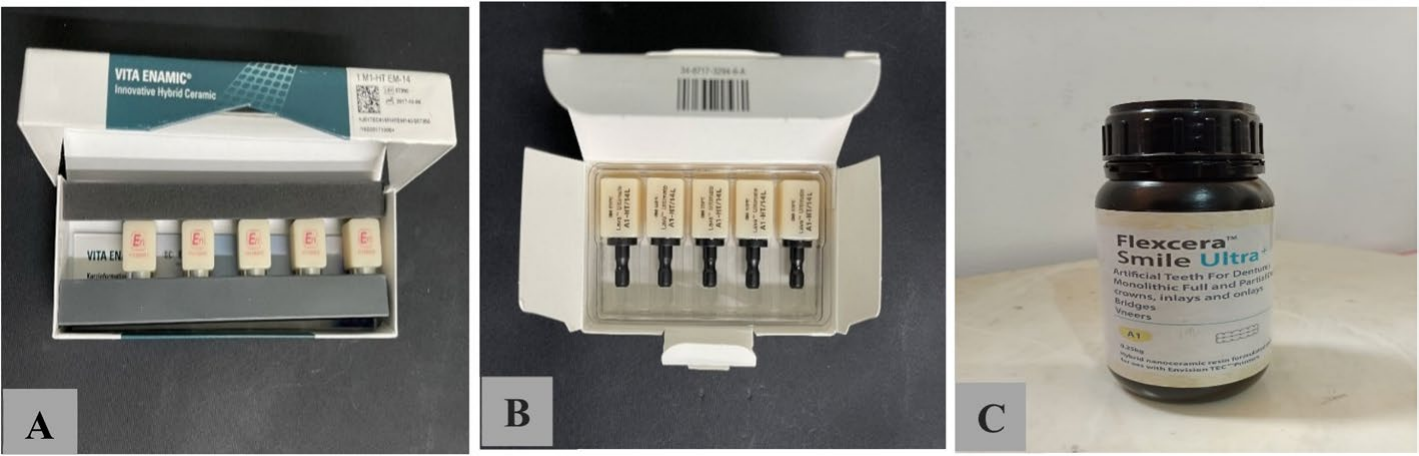
The materials used in the study, **A** Vita Enamic Blocks, **B** Lava Ultimate Blocks, **C** Flexcera Smile Ultra^+^ bottle

The materials used in the current study are shown in Table 1 and Fig. 1.

**Table 1:** Materials used in the study

**Material**	**Description**	**Composition**	**Manufacturer**	**Shade and Translucency**	**Lot No**
Vita Enamic® (VE)	Block Size: EM-14	-14 wt. % UDMA, TEGDMA - 86 wt.% Feldspar ceramic enriched with aluminum oxide [20]	Vita Zahnfabrik, Bad Sackingen, Germany	1M1, HT	57,350
Lava™ Ultimate (LU)	Block Size: 14L	- 20 wt. % Bis-GMA, UDMA, Bis-EMA, TEGDMA - 80 wt. % SiO2 (20 nm), ZrO2 (4–11 nm), ZrO2 /SiO2 clusters [20]	3M ESPE, Saint Paul, MN, USA	A1, HT	N946038
Flexcera™ Smile Ultra + (FSU +)	Liquid hybrid nanoceramic resin	Diphenyl(2,4,6-trimethylbenzoyl) phosphine oxide, methacrylate monomer, methacrylic oligomer & inorganic nanofillers [21]	Desktop Health, Newport Beach, California, USA	A1	310122b

The devices used in the current study are shown in Table 2.

**Table 2:** Devices used in the study

Device name	Manufacturer
Low speed water-cooled diamond microsaw (Fig. 2A)	Isomet 4000, Buehler, Germany
Digital light processing 3D printer (Fig. 4A)	Mogassam, Cairo, Egypt
Light-curing unit (Fig. 5)	Mogassam, Cairo, Egypt
Fourier transform infrared spectrophotometer (Fig. 6)	IRPrestige-21, SHIMADZU, Japan
Contact surface roughness styler (Fig. 7)	Mitutoyo SJ-210, Japan
Universal testing machine (Fig. 8)	Instron 2519, UK
Scanning electron microscope (Fig. 10)	Quanta 3D 200i, FEI Company, The Netherlands

## Methods

### Preparation and grouping of specimens

One-hundred and twenty-five (125) bar-shaped specimens of the three different hybrid resin materials were used in this study, being classified into 5 groups (n = 25). Group I: Lava Ultimate (LU), group II: Vita Enamic (VE), group III: Flexcera Smile Ultra^+^ A (FSU^+^ A), group IV: Flexcera Smile Ultra^+^ B (FSU^+^ B) and group V: Flexcera Smile Ultra^+^ C (FSU^+^ C). In addition, extra three un-cured specimens (n = 1/ FSU^+^ grp) were used.

**Fig. 2:**
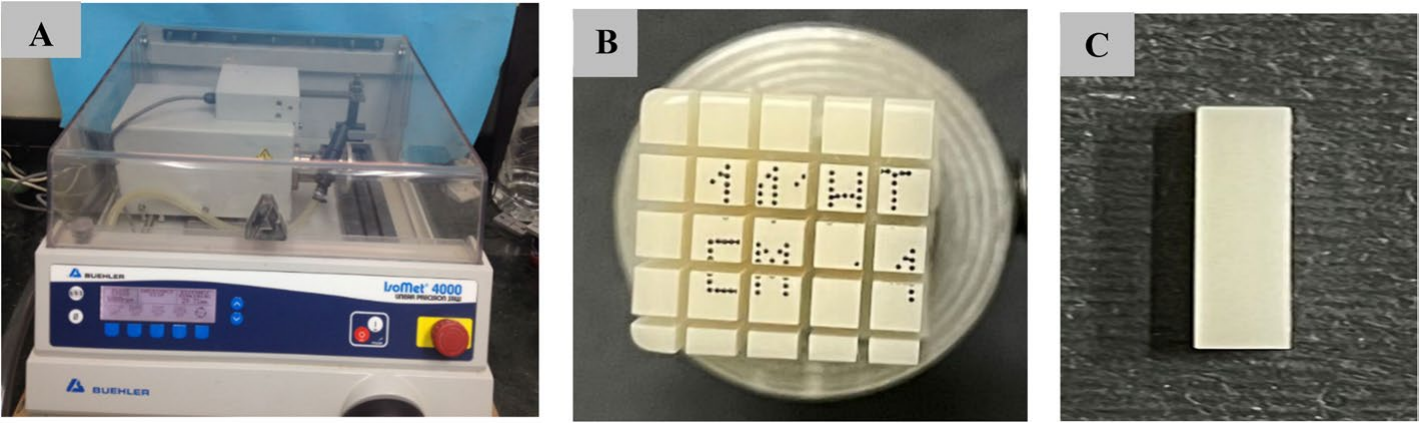
**A** Low-speed water-cooled diamond microsaw used in the study, **B** CAD/CAM block after cutting, **C** Bar-shaped specimen (14 × 2x2 mm)

**Fig. 3:**
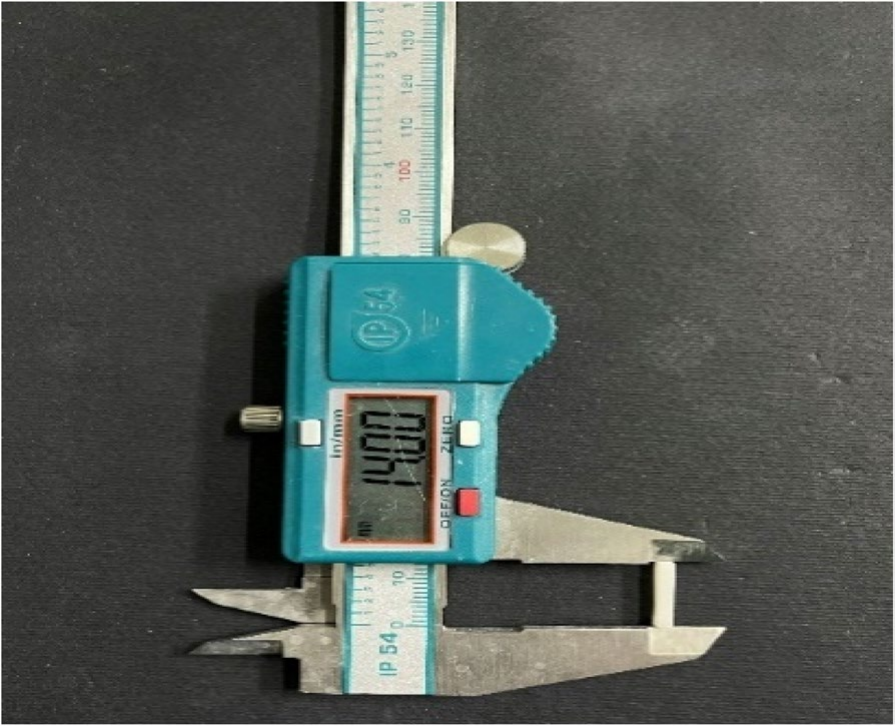
Digital caliper used in the study

A low-speed water-cooled diamond microsaw (Isomet 4000, Buehler, Germany) (Fig. 2A) was used to cut the CAD/CAM blocks (LU and VE blocks) to have bar-shaped specimens (n = 25), 14 mm length, 2 mm width and 2 mm thickness (Fig. 2B and 2C). A digital caliper (TMT321506 IP54, Micro Ohm Electronics, Cairo, Egypt) was used to ensure specimens’ dimensions (Fig. 3), which kept dry at room temperature after being finished with 220 grit wet silicon carbide (3 M ESPE, Saint Paul, MN, USA) [1]. Because of the size of the material blocks, specimens could not be prepared according to ISO 4049 [22].

**Fig. 4:**
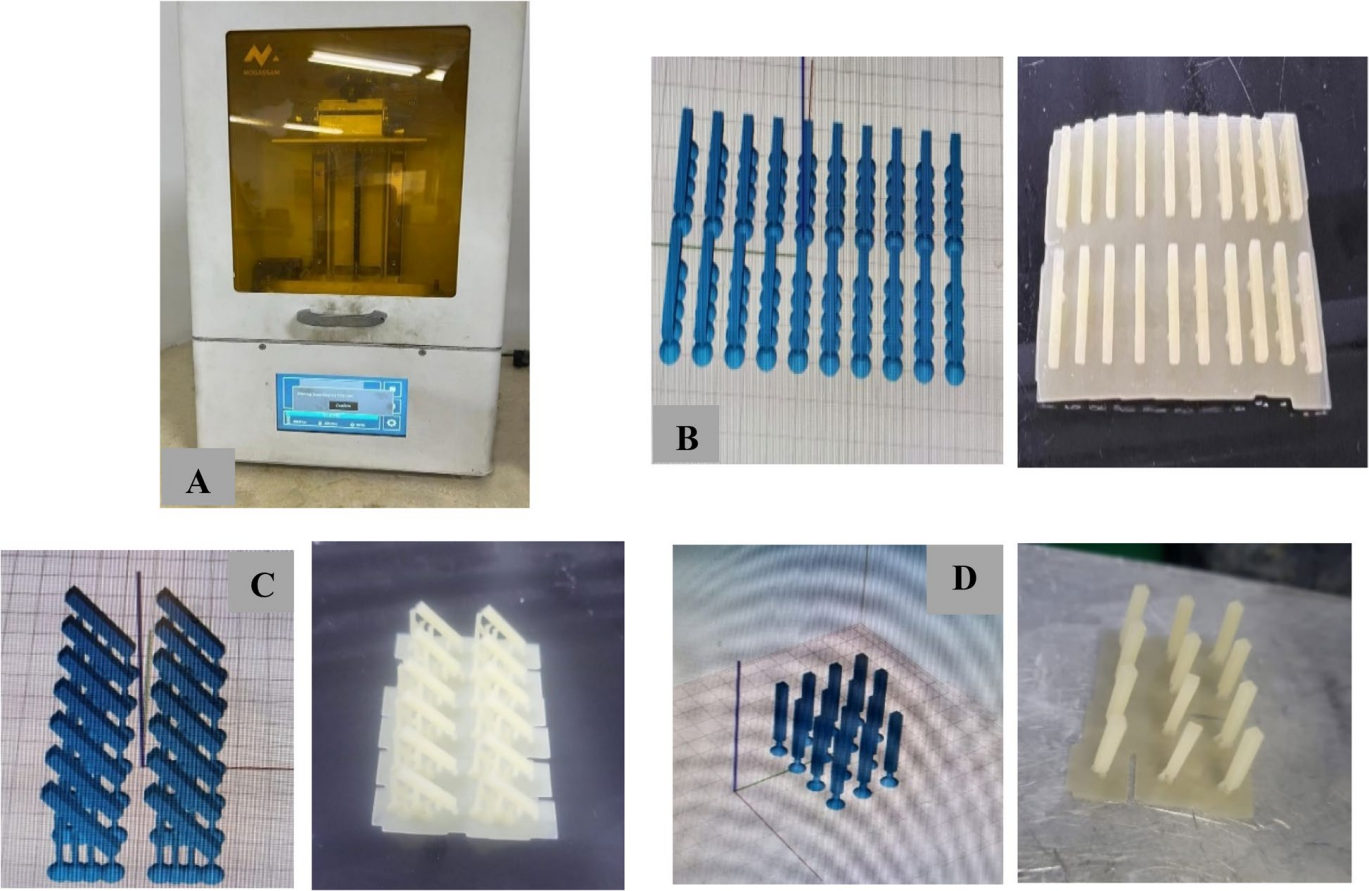
**A** DLP 3D printer used in the study, **B** Bar-shaped specimens printed with an angle of 0-degree (software and virtual photos), **C** Bar-shaped specimens printed with an angle of 45-degree (software and virtual photos), **D** Bar-shaped specimens printed with an angle of 90 degree (software and virtual photos)

Three groups of FSU^+^ bar-shaped specimens (14 mm length, 2 mm width, and 2 mm thickness) (n = 25), were 3D printed using a digital light processing (DLP) 3D printer (Mogassam, Cairo, Egypt) (Fig. 4A), using a Chitubox V1.9.1 software. The group FSU^+^ A specimens were printed with an angle of 0 degree (in a horizontal direction, parallel to the platform) (Fig. 4B), while the group FSU^+^ B specimens were printed with an angle of 45 degree to the platform (Fig. 4C), and the group FSU^+^ C specimens were printed with an angle of 90 degree (in a vertical direction, perpendicular to the platform) (Fig. 4D).

**Fig. 5:**
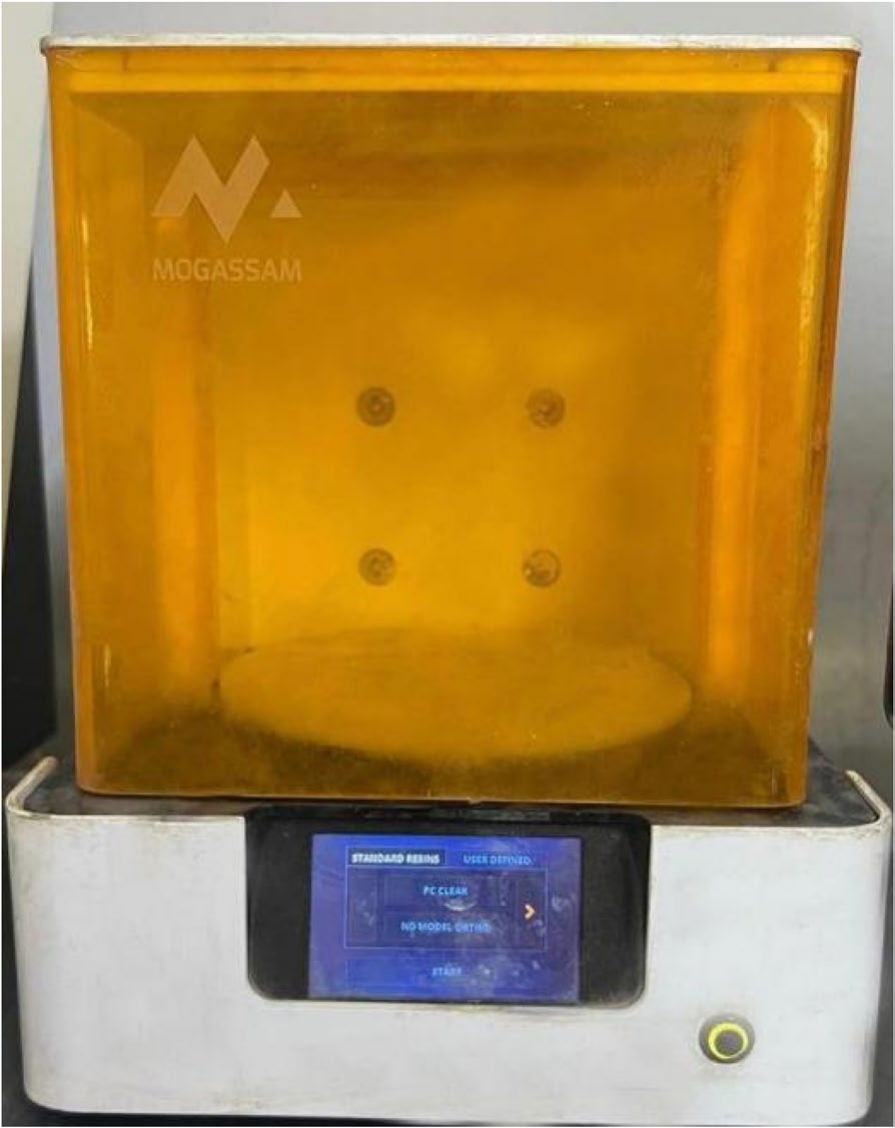
Light-curing unit used in the study

For FSU^+^ hybrid resin material, the resin profile has been optimized to have 5 mm for the lift distance, 60 mm/s for the lift speed, 0.05 mm for the layer height, 8 for the bottom layer count, 6.5 for the exposure time, and 20 for the bottom exposure [1, 23]. Afterwards, the printed specimens were properly cleaned with 99% ethyl alcohol according to the manufacturer’s instructions, then exposed to a light-curing unit (Mogassam, Cairo, Egypt) (Fig. 5) twice for 45 min, rotating the specimens following initial exposure. Finally, the specimens were finished with wet silicon carbide, and a digital caliper was used again to ensure correct specimens’ dimensions [1].

### Characterization of the new 3D-printed material, measuring its degree of conversion

A total of eighteen (18) bar-shaped specimens were tested: comprising the extra three uncured specimens (n = 1/ FSU^+^ grp) and fifteen cured specimens (n = 5/ FSU^+^ grp). Using a mortar and pestle, each specimen was manually ground into fine powder to be mixed with potassium bromide (KBr) in a ratio of 2:200 mg respectively. Both powders were then compressed into disk form.

**Fig. 6:**
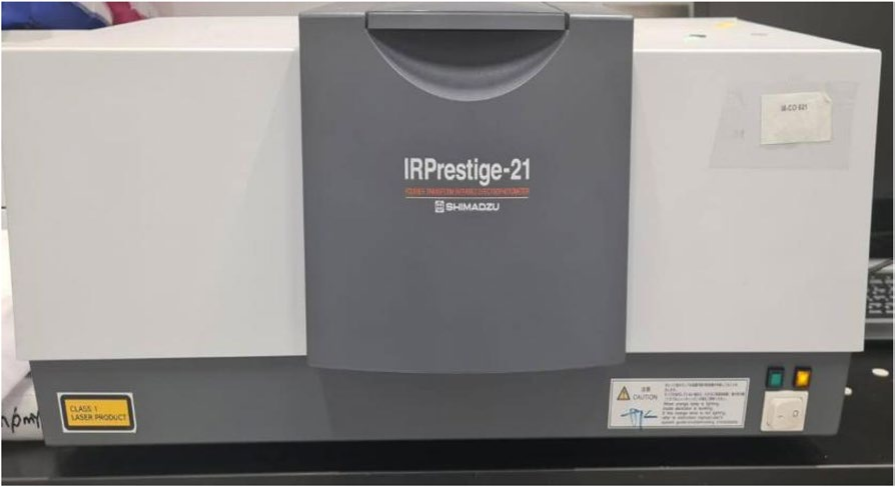
Fourier transform infrared spectrophotometer (FTIR) used in the study

The compressed disk was irradiated by the infrared spectrum of the Fourier transform infrared spectrophotometer (FTIR) (IRPrestige-21, SHIMADZU, Japan) (Fig. 6), using IRsolution software. The material's absorbance ratio was measured using the following parameters: 400 to 4000 cm^−1^ wavelengths, 20 scans, and 4 cm^−1^ resolution. IR absorbance peaks cause stretching vibrations of the aliphatic C = C double bonds at 1716.6 to 1732.08 cm^−1^ and aromatic C….C double bonds (as internal reference) at 1450.47 to 1527.62 cm^−1^ [24, 25]. The percentage of degree of conversion (DC %) was calculated using the following equation [26]:

$$\text{DC \% }= \{1- (\text{C}=\text{C}/\text{C}...\text{C})\text{ cured}/(\text{C}=\text{C}/\text{C}...\text{C})\text{ un}-\text{cured}\} \times 100$$

### Surface roughness testing

**Fig. 7:**
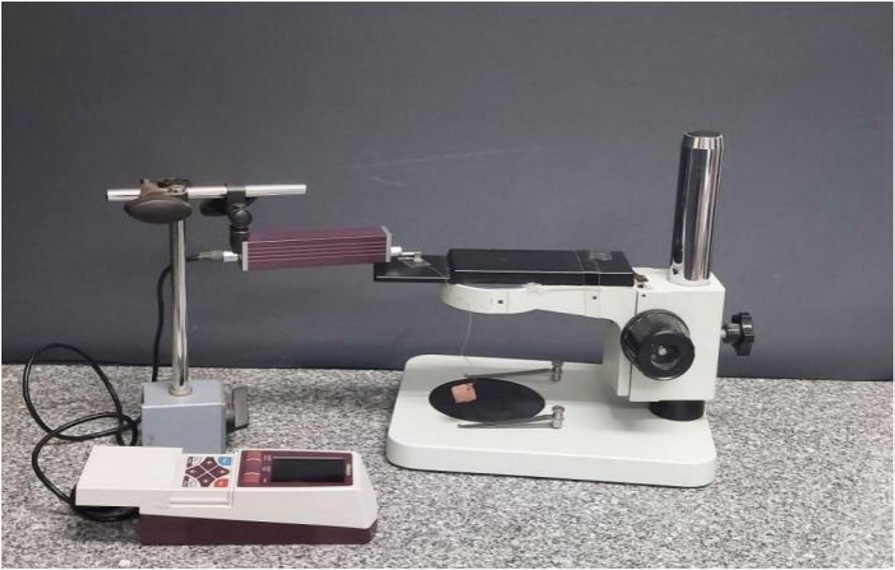
Contact surface roughness styler used in the study

A total of fifty (50) bar-shaped specimens (n = 10/grp) were tested. The surface roughness values were measured using a contact surface roughness styler (Mitutoyo SJ-210, Japan) (Fig. 7). Prior to measurement, the device was calibrated to achieve the standard calibration (12 mm measuring distance, 0.5 mm/s measuring speed and 0.75 mN measuring force) according to ISO 21920–2 [27]. Three readings were recorded for each specimen at a distance 500 µm each, using a stylus having the following properties (tip radius of 2 µm, and tip angle of 60 degree).

### Flexural strength (FS) and Flexural modulus (FM) testing

**Fig. 8:**
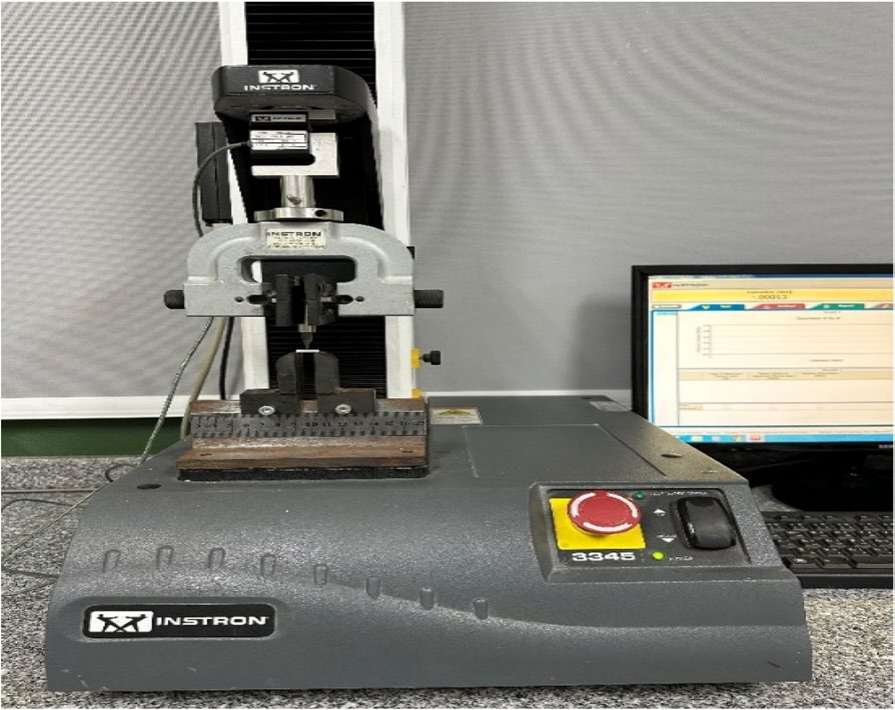
Universal testing machine used in the study

A total of fifty (50) bar-shaped specimens (n = 10/grp) were tested. A three-point bending test was used to measure the flexural strength (FS) and flexural modulus (FM). The test was carried out using a universal testing machine (Instron 2519, UK) (Fig. 8) with a 12 mm support span and a 1 mm/min cross-head speed with a BlueHill universal software (Instron, UK) [28]. The following equations were used to calculate FS and FM:

$$\begin{array}{cc}\text{FS}= 3\text{Fl }/ {2\text{bh}}^{2}& \text{FM }= {\text{Fl}}^{3}/ {4\text{bh}}^{3}\text{d}\end{array}$$

where F is the maximum load (N), l is the distance between supports = 12 mm, b is the specimen width (mm), h is the specimen height (mm) and d is the deflection at the end of the linear part of the force–deflection diagram (mm).

### Fractographic analysis

**Fig. 9:**
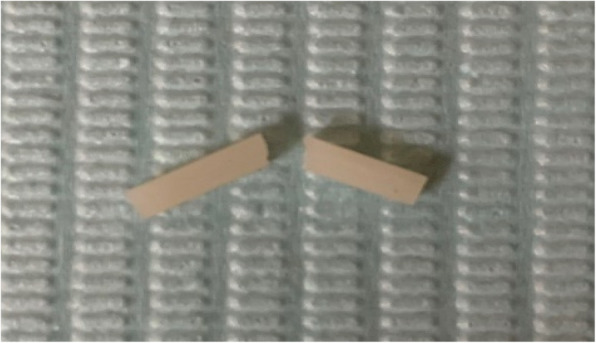
Fractured flexure test specimen used for the fractographic analysis

**Fig. 10:**
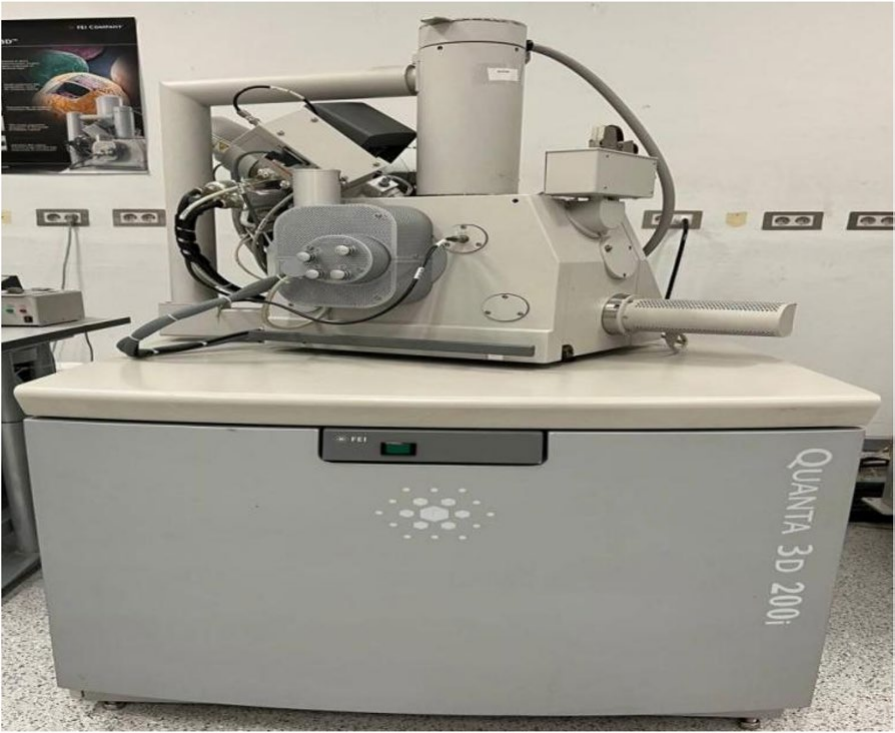
Scanning electron microscope (SEM) used in the study

A total of twenty-five (25) fractured specimens (n = 5/grp) were tested (Fig. 9) without application of a coating layer to their surfaces. A scanning electron microscope (SEM) (Quanta 3D 200i, FEI Company, The Netherlands) (Fig. 10) was used at magnifications 100 × up to 2000x. An accelerating voltage of 20 kv under low vacuum, large field electron (LFED) and back scattered electron (BSED) detectors with working distance of 15–17 mm were used during the SEM investigation.

### Statistical analysis and data interpretation

SPSS software, version 25 (SPSS Inc., PASW statistics for windows version 25. Chicago: SPSS Inc.) was used to analyze data. Qualitative data were described using numbers and percentages. Quantitative data were described using mean ± standard deviation for normally distributed data after testing normality using Shapiro Wilk test. The results significance was judged at p ≤ 0.05 level. One Way ANOVA test was used to compare more than 2 independent groups with Post Hoc Tukey test to detect pairwise comparisons.

## Results

### Degree of conversion (DC%) of the three Flexcera Smile Ultra^+^***(FSU***^+^***) ***groups

**Fig. 11:**
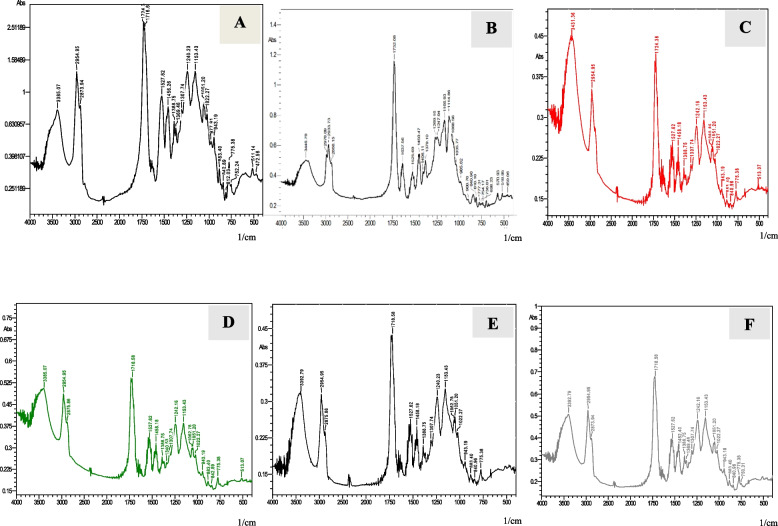
FTIR analysis for un-cured specimen of FSU^+^A group (**A**), FTIR analysis for five cured specimens of FSU.^+^A group (**B**,** C**,** D**,** E **and** F**)

The absorbance peaks of uncured and cured specimens for FSU^+^A group (printed with an angle of 0 degree) are shown in (Fig. 11). For the uncured specimen, at wavelength 1716.6 cm^−1^, the absorbance peak of the aliphatic C = C double bond is 2.51, whereas the internal reference C….C at wavelength 1527.62 cm^−1^ is 1. The absorbance peaks of five cured specimens’ aliphatic C = C double bonds at wavelengths 1718.58 to 1732.08 cm^−1^ are 1.16, 0.42, 0.56, 0.44 and 0.68, while the internal reference C….C at wavelengths 1450.47 to 1527.62 cm^−1^ are 0.56, 0.26, 0.32, 0.26 and 0.42 respectively.

**Fig. 12:**
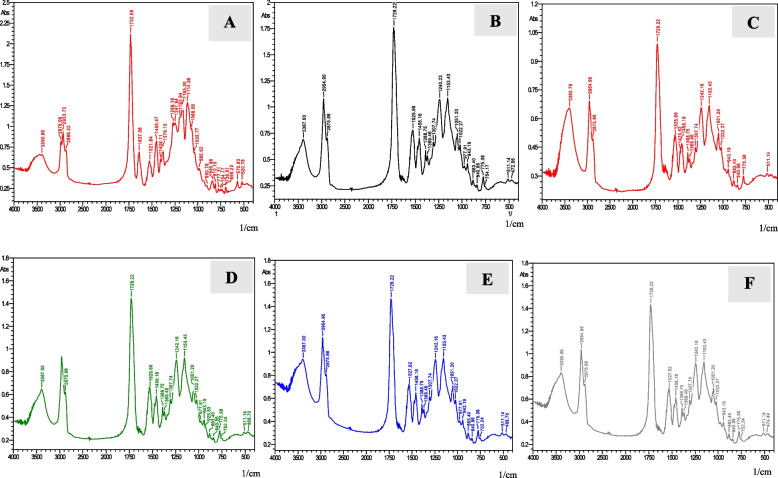
FTIR analysis for un-cured specimen of FSU^+^B group (**A**), FTIR analysis for five cured specimens of FSU^+^B group (**B**,** C**,** D**,** E **and** F**)

The absorbance peaks of uncured and cured specimens for FSU^+^ B group (printed with an angle of 45 degree) are shown in (Fig. 12). For the uncured specimen, at wavelength 1732.08 cm^−1^, the absorbance peak of the aliphatic C = C double bond is 2.15, whereas the internal reference C….C at wavelength 1521.84 cm^−1^ is 0.55. The absorbance peaks of five cured specimens’ aliphatic C = C double bonds at wavelength 1728.22 cm^−1^ are 1.75, 1.02, 1.48, 1.48 and 1.44, while the internal reference C…….C at wavelengths 1525.69 to 1527.62 cm^−1^ are 0.75, 0.54, 0.72, 0.72 and 0.68 respectively.

**Fig. 13:**
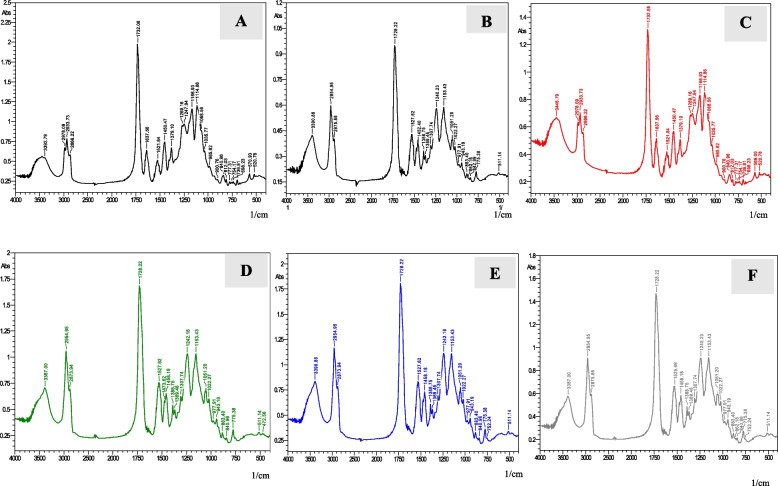
FTIR analysis for un-cured specimen of FSU^+^C group (**A**), FTIR analysis for five cured specimens of FSU^+^C group (**B**,** C**,** D**,** E **and** F)**

The absorbance peaks of uncured and cured specimens for FSU^+^C group (printed with an angle of 90 degree) are shown in (Fig. 13). For the uncured specimen, at wavelength 1732.08 cm^−1^, the absorbance peak of the aliphatic C = C double bond is 2, whereas the internal reference C….C at wavelength 1521.84 cm^−1^ is 0.55. The absorbance peaks of five cured specimens’ aliphatic C = C double bonds at wavelengths 1728.22 to 1732.08 cm^−1^ are 0.93, 1.32, 1.7, 1.8 and 1.48, while the internal reference C….C at wavelengths 1450.47 to 1527.62 cm^−1^ are 0.42, 0.56, 0.75, 0.85 and 0.64 respectively.

**Fig. 14:**
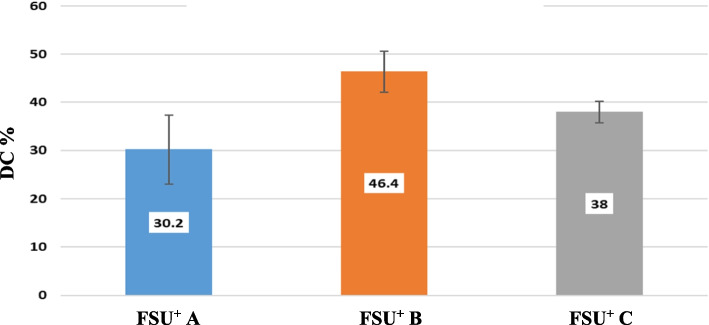
Mean and standard deviation (SD) values of DC % for the tested groups

FSU^+^ B group showed the highest mean DC% value (46.40 ± 4.28). On the other hand, FSU^+^ A group showed the lowest mean DC% value (30.20 ± 7.12). Post Hoc Tukey test demonstrated a statistically significant difference among tested groups (p ≤ 0.05). Mean and standard deviation (SD) values of DC% for the tested groups are represented in Table 3 and Fig. 14.

**Table 3:** Mean and standard deviation (SD) values of DC % for the tested groups

**Degree of conversion**	**FSU**^**+**^** A** **(0 degree)**	**FSU**^**+**^** B** **(45 degree)**	**FSU**^**+**^** C** **(90 degree)**	**Test of significance**
Mean ± SD	30.20 ± 7.12^a^	46.40 ± 4.28^b^	38.0 ± 2.24^c^	F = 13.31 P = 0.001*

F: One Way ANOVA test, * statistically significant

Similar superscripted letters denote insignificant difference between studied groups within same row by Post Hoc Tukey test

### Surface roughness testing

**Fig. 15:**
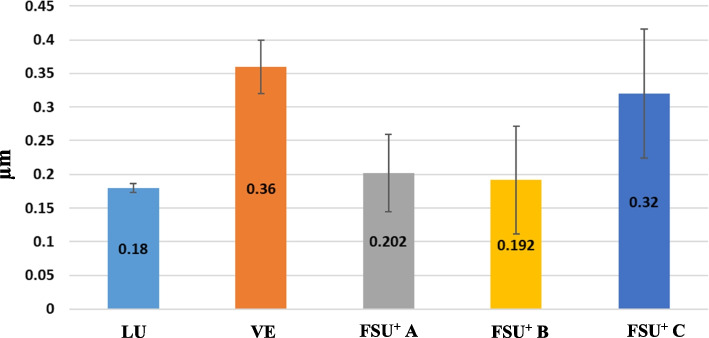
Mean and standard deviation (SD) values of surface roughness (µm) for the tested groups

The surface roughness from higher to lower values can be sorted as VE > FSU^+^ C > FSU^+^ A > FSU^+^ B > LU. VE group showed the highest mean surface roughness value (0.360 ± 0.04), while LU group showed the lowest mean surface roughness value (0.180 ± 0.007). Post Hoc Tukey test demonstrated a statistically significant difference (p ≤ 0.05) between LU and VE groups, LU and FSU^+^ C groups, VE and FSU^+^ A groups as well as VE and FSU^+^ B groups. Furthermore, there was a statistically significant difference (p ≤ 0.05) between FSU^+^ A and FSU^+^ C groups on one hand, and FSU^+^ B and FSU^+^ C groups on the other hand. Mean and standard deviation (SD) values of surface roughness for the tested groups are represented in Table 4 and Fig. 15.

**Table 4:** Mean and standard deviation (SD) values of surface roughness (µm) for the tested groups

**Surface roughness**	**LU**	**VE**	**FSU**^**+**^** A** **(0 degree)**	**FSU**^**+**^** B** **(45 degree)**	**FSU**^**+**^** C** **(90 degree)**	**Test of significance**
Mean ± SD	0.180 ± 0.007^ab^	0.360 ± 0.04^c^	0.202 ± 0.057^bd^	0.192 ± 0.08^bd^	0.32 ± 0.096^c^	F = 8.51 P < 0.001*

F: One Way ANOVA test, * statistically significant

Similar superscripted letters denote insignificant difference between studied groups within same row by Post Hoc Tukey test

### Flexural strength (FS) and Flexural (FM) modulus

The flexural strength from higher to lower values can be sorted as LU > VE > FSU^+^ B > FSU^+^ C > FSU^+^ A. LU group showed the highest mean FS value (169.87 ± 29.69), while FSU^+^ A group showed the lowest mean FS value (78.14 ± 14.56). Post Hoc Tukey test demonstrated a statistically significant difference (p ≤ 0.05) among tested groups except between FSU^+^ A and FSU^+^ C groups as well as between FSU^+^ B and FSU^+^ C groups.

**Fig. 16:**
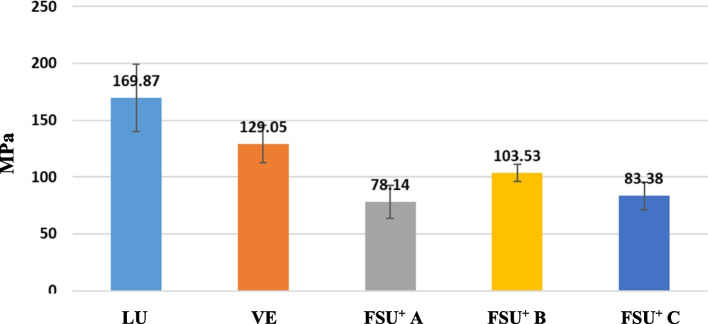
Mean and standard deviation (SD) values of FS (MPa) for the tested groups

**Fig. 17:**
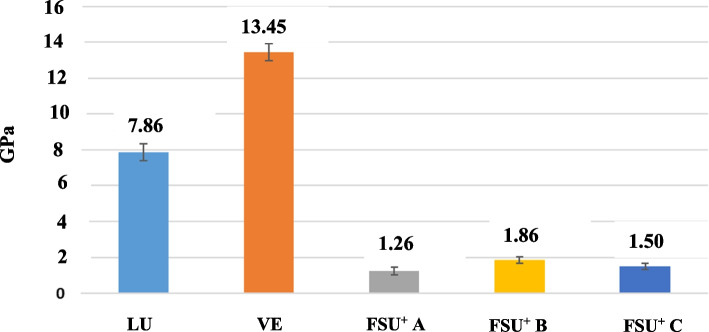
Mean and standard deviation (SD) values of FM(GPa) for the tested groups

The flexural modulus from higher to lower values can be sorted as VE > LU > FSU^+^ B > FSU^+^ C > FSU^+^ A. VE group showed the highest mean FM value (13.45 ± 0.47), while FSU^+^ A group showed the lowest mean FM value (1.26 ± 0.22). Post Hoc Tukey test demonstrated a statistically significant difference (p ≤ 0.05) among tested groups except between FSU^+^ A and FSU^+^ C groups as well as between FSU^+^ B and FSU^+^ C groups. Mean and standard deviation (SD) values of FS and FM for the tested groups are represented in Table 5 and Figs. 16 and 17.

## Fractographic analysis

**Table 5:** Mean and standard deviation (SD) values of FS (MPa) and FM (GPa) for the tested groups

**Group** **Test**	**LU**	**VE**	**FSU + A** **(0 degree)**	**FSU**^**+**^** B** **(45 degree)**	**FSU + C** **(90 degree)**	**Test of significance**
	Mean ± SD	Mean ± SD	Mean ± SD	Mean ± SD	Mean ± SD	
**FS**	169.87 ± 29.69^a^	129.05 ± 16.40^b^	78.14 ± 14.56^c^	103.53 ± 7.77^d^	83.38 ± 12.27^cd^	F = 22.54 P < 0.001*
**FM**	7.86 ± 0.47^a^	13.45 ± 0.47^b^	1.26 ± 0.22^c^	1.86 ± 0.16^d^	1.50 ± 0.17^cd^	F = 22.54 P < 0.001*

F: One Way ANOVA test, * statistically significant

Similar superscripted letters denote insignificant difference between studied groups within same row by Post Hoc Tukey test

**Fig. 18:**
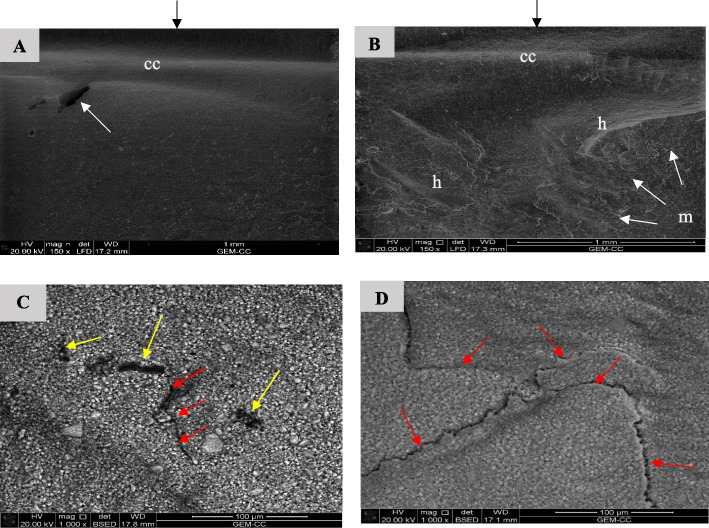
SEM examination of fractured surfaces of LU specimens. Black arrows on the top of images A and B indicate load direction. **A** SEM image showing compression curl (cc) and surface void (white arrow) [magnification 150x, LFED], **B** SEM image showing fracture mirror (m), hackle lines (h) and compression curl (cc). White arrows indicate the origin and direction of crack propagation [magnification 150x, LFED], **C** SEM image showing fracture origin at surface voids (yellow arrows) and direction of crack propagation (spreading from a surface void along the particles boundaries) (red arrows) [magnification 1000 x, BSED], **D** SEM image showing direction of crack propagation (spreading along the particles boundaries) (red arrows) [magnification 1000 x, BSED]

**Fig. 19:**
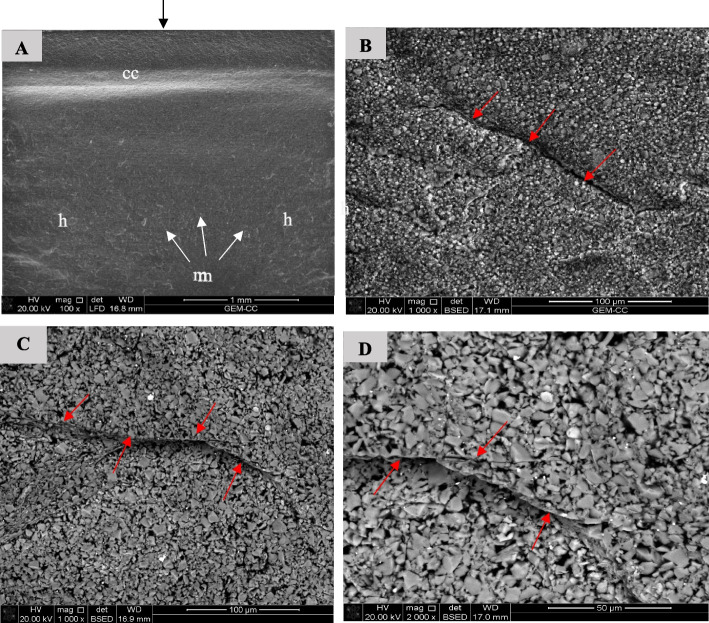
SEM examination of fractured surfaces of VE specimens. Black arrow on the top of image A indicates load direction. **A** SEM image showing fracture mirror (m), hackle lines (h) and compression curl (cc). White arrows indicate the origin and direction of crack propagation [magnification 100x, LFED], **B** SEM image showing direction of crack propagation (red arrows) [magnification 1000 x, BSED], **C** SEM image showing direction of crack propagation (red arrows) [magnification 1000 x, BSED], **D** SEM image showing direction of crack propagation (red arrows) [magnification 2000 x, BSED]

**Fig. 20:**
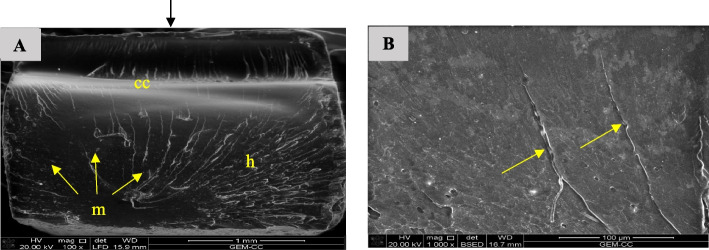
SEM examination of fractured surfaces of FSU^+^A specimens. Black arrow on the top of image A indicates load direction. **A** SEM image showing fracture mirror (m), hackle lines (h) and compression curl (cc). Yellow arrows indicate the origin and direction of crack propagation [magnification 100x, LFED], **B** SEM image showing magnification of hackle lines (yellow arrows) [magnification 1000 x, BSED]

**Fig. 21:**
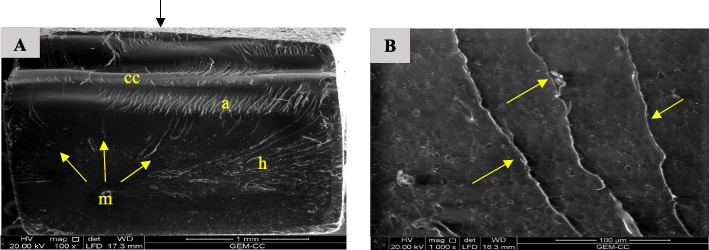
SEM examination of fractured surfaces of FSU^+^B specimens. Black arrow on the top of image A indicates load direction. **A** SEM image showing fracture mirror (m), arrest lines (a), hackle lines (h) and compression curl (cc). Yellow arrows indicate the origin and direction of crack propagation [magnification 100x, LFED], **B** SEM image showing magnification of hackle lines (yellow arrows) [magnification 1000 x, BSED]

**Fig. 22:**
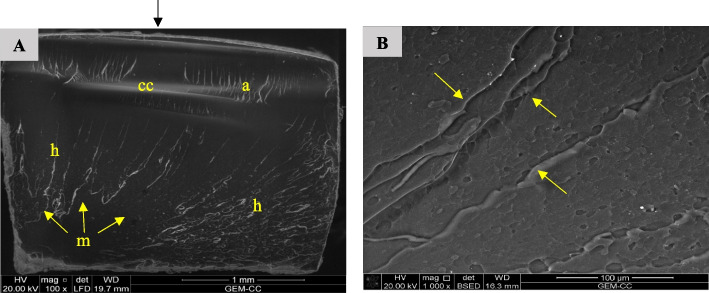
SEM examination of fractured surfaces of FSU^+^ C specimens. Black arrow on the top of image A indicates load direction. **A** SEM image showing fracture mirror (m), arrest lines (a), hackle lines (h) and compression curl (cc). Yellow arrows indicate the origin and direction of crack propagation [magnification 100x, LFED], **B** SEM image showing magnification of hackle lines (yellow arrows) [magnification 1000 x, BSED]

The SEM inspection of the fractured bar-shaped specimens revealed a compression curl in the upper part and the fracture origin in the lower portion of the surface, which denotes specimens bending. A higher magnification showed microcracks in the fracture structure, locating crack initiation sites such as voids, as well as arrest lines and hackle lines (Fig. 18, 19, 20, 21 and 22). Fractured LU and VE specimens showed different crack propagation behaviors (Figs. 18D, 19C and D).

## Discussion

Great interest has been focused on AM, especially expanding the use of digital dental applications. CAD software carefully analyze the data, to produce a 3D model, followed by the CAM software which by transferring the design to milling or 3D printing machines, final dental prosthetics are produced. 3D printing technology can be used to fabricate different appliances such as dental crowns and bridges [24].

SLA and DLP are the two main 3D printing technologies used in dentistry. Compared to standard SLA, DLP 3D printing offers the advantage of faster printed layer fabrication, enabling the print and cure of a single layer throughout the whole build platform in few seconds. DLP also has the advantage of using less material than SLA and other 3D printing techniques, which lowers production costs [24]. However, because of the layered fabrication technique, mechanical anisotropy and low filler content are the major disadvantages of AM [23].

Additive technology is frequently utilized to fabricate dental crowns from polymer-based and hybrid resin materials. However, further studies are still required to evaluate their use in terms of degree of conversion, fractography, as well as mechanical and surface properties [24]. Flexcera Smile Ultra Plus (Desktop Health, Newport Beach, California, USA) is an example of a newly introduced 3D printable light-cured hybrid nanoceramic resin material with very limited studies conducted on it. It can be used for the construction of dentures’ artificial teeth, monolithic full and partial dentures, single crowns, bridges, inlays, onlays and veneers [29].

Using the subtractive (milling) method of CAD/CAM offers many benefits to the dental filed. A digital scan with a milling machine removes traditional impressions, diagnostic casts, wax-ups, investing, casting, and firing [30].

Milling machines allow single visit final restorations, thus eliminate the step of temporary crown fabrication, finally being more comfortable to patients. Especially, that multi-axis milling machines provide accurate and rapid prosthesis [31]. High cost and the accuracy of the prosthesis, possibly being affected by the wear of milling burs along with the limitation of the bur’s path are considered its main drawbacks [32].

Clinically, Vita Enamic (Vita Zahnfabrik, Bad Sackingen, Germany), and Lava Ultimate (3 M ESPE, Saint Paul, MN, USA) are commonly used millable hybrid resin blocks. Lava Ultimate is a resin loaded with nanoparticles and nanoclusters, while Vita Enamic is a polymer-infiltrated ceramic-network (PICN) dental material. Proper evaluation of their properties is necessary to better understand the advantages and disadvantages [20]. Therefore, further studies are still needed, especially comparing them with 3D printed hybrid resin materials.

The printing process can be affected by about 50 factors [33]. Most factors, such as the printer, printing parameters, layer thickness and the postprocessing, are provided by the manufacturers. Nevertheless, there are very few suggestions on the printing angle and orientation in the printing software. So, to evaluate the performance of a restoration it is important to examine the effects of printing angle and orientation on its properties [23].

As the DC of a material greatly influences its mechanical properties, it needs to be evaluated [34]. Despite the fact that there is no parameter can aid in predicting the material’s clinical endurance and success or failure. Features like flexural strength and flexural modulus can give clues about how these materials will behave under occlusal stresses [35].

High flexural strength of a restoration is required to withstand masticatory forces [36]. Furthermore, fractographic examination of the fractured specimens allows better understanding of the fracture mechanism [23]. Surface roughness may result in dental caries along with severe wear to the antagonist tooth, causing unstable occlusion [37].

The null hypothesis was rejected because of significant differences in DC, mechanical & surface properties and fractographic examination between hybrid resin materials for printing and for milling.

Because of the size of the material blocks (LU and VE blocks), specimens could not be prepared according to ISO 4049 which outlines specific specimen dimensions (typically 2mm x 2mm x 25mm) for flexural strength and testing procedures. Deviating from these dimensions can lead to inaccurate flexural strength and modulus values making it difficult to compare the material's performance with other materials tested according to the standard [22].

Although the degree of conversion may be measured by different techniques such as electron paramagnetic resonance, nuclear magnetic resonance, differential scanning calorimetry and differential thermal analysis, FTIR remains the most frequently used technique, measuring the C = C stretching vibrations both before and after curing of materials. Besides being verified as a standard technique, it is also a reliable method due to the availability of equipment [38–40].

The layering technique used in AM technology could lead to insufficient polymerization per layer which might finally decrease the degree of polymerization. Hence, the post-curing process was introduced to convert the unreacted monomers into polymers with increased DC [41]. An increased DC usually improves the physical, mechanical, chemical, and biological properties of light-cured resin materials [42, 43]. The outcomes of the present study showed a significant difference in DC values between FSU^+^ three groups printed with different angles. This finding agrees with a previous study that illustrated a strong relation between the printing angle (orientation) and DC [44].

In this earlier study, the specimens printed with 45- and 90-degree angles showed higher DC values than those printed with 0-degree angle which agrees with the present study. However, specimens printed with 90-degree angle showed higher DC values compared to those printed with 45-degree angle, disagreeing with our results. This may be due to the difference in materials’ composition, printing parameters, types of DLP printers, and post curing units [44]. According to this earlier study, different number of printed layers with different cross sections at different printing angles may be an acceptable explanation. Increased number of layers with smaller cross sections leads to profound light exposure with subsequent higher DC [44].

Surface roughness is considered to be one of the most crucial characteristics that can impact the life span of dental prostheses [45]. More understanding of the surface topography of AM materials is required because of the nature and technique of multilayer printing [46, 47]. The surfaces of printed items were affected by changes of the printing conditions and parameters. Furthermore, the layering direction in relation to specimens’ surfaces might have an impact on the surface characteristics [46]. It was claimed that the morphological characteristics and porosity of printed items are affected by printing angles and orientations [48, 49].

The present study used a contact surface roughness styler to measure specimens’ surface roughness using a small load (0.75 mN). Although the contact stylus technique may damage surfaces in case of high loads, it is recommended by the ISO [50]. This technique provides reliable measurements because the styler directly touches the specimen and it is able to measure long distances [51].

Our results declared variations in surface roughness values between different groups. These variations may be attributed to individual variations in specimens’ polishing and fillers’ size. There was a significant difference in surface roughness values between VE and LU (VE surface roughness value > LU surface roughness value). This finding agrees with previous studies, which stated the VE being an interpenetrating phase ceramic composed of a porous ceramic core infiltrated with resin. Thus, the weaker polymer matrix might be easily separated from the ceramic network, resulting in higher roughness values [52, 53].

Furthermore, there was a significant increase in surface roughness value of FSU^+^ C group (printed with an angle of 90 degree) when compared to that of FSU^+^ A and FSU^+^ B. According to previous studies, this finding may be due to different numbers of printing layers with different numbers of steps between adjacent layers [54, 55]. It also may be due to the impact of different printing orientations on surface porosity and morphological characteristics of printed objects as mentioned before [48, 49].

In a three-point bending test, a shorter support span (12 mm) relative to the specimen length (14 mm) focuses the applied load to generate a clear bending failure with a measurable region of the specimen making it easier and more accurate to measure the deflection of the specimen at the loading point (usually the midpoint between the supports). In addition, the longer ends of the specimen provide stable reference points for deflection measuring devices [56].

The presented results showed that the recorded FS values of LU and VE are nearly comparable to the results of an earlier study [57]. It is possible to explain this slight difference by little variations in the block production method and/or by variable specimen manufacturing [1]. LU group showed significantly higher FS values compared to other groups, being significantly higher than those of VE group. This finding agrees with a previous study [57]. This may be because interfacial adhesion is more difficult to accomplish when resin infiltrates the sintered porous ceramic matrix [58, 59].

Despite FS being the fundamental metric that highlights the variations between the materials, it is overestimated in its significance. The material's stiffness and how it behaves when a load is applied are not revealed by the strength. The FM is typically assessed to learn more about the strain–stress characteristics of the materials being examined [60].

The VE group showed significantly higher FM values than those of other groups, agreeing with previous studies which stated that the reason may be the high loading (86 wt.%) and ceramic skeleton of PICN materials [1, 60].

The low filler content of FSU^+^ may be the cause of the lower FS/FM values of FSU^+^ groups when compared to VE and LU values. To maintain the liquid consistency during the 3D printing process, a relatively low filler content is required [1].

The FSU^+^ B group (printed with an angle of 45 degree) showed higher FS/FM values compared to those of the FSU^+^ A and FSU^+^ C groups. This could be because of its higher degree of conversion, which enhances mechanical properties. This finding is in accordance with an earlier study which stated that, the printed items ought to be positioned so that the tensile force produced during mastication can be applied along the layers rather than across them [1].

Bending of the fractured specimens is indicated by the presence of compression curl in the upper part and the fracture origin in the lower portion of the surface. LU specimens' fracture surfaces showed crack deflection as a result of particles bypassing, resulting in elongation of the fracture path, which is associated with higher fracture energy consumption [1]. Crack bridging by filler particles was observed in the fracture surface of the VE specimens consuming high energy. This finding agrees with a previous study [1].

Hackle lines observed in the fracture surface of FSU^+^ specimens represent the crack pathway as well as indicate rapid crack propagation [61]. This finding may be because of the low filler content of FSU^+^ 3D printed material.

Although our study provided valuable insights into the three different hybrid resin materials’ behavior, some limitations should be taken into consideration such as the specimen dimensions (14 mm × 2 mm × 2 mm) which may affect the accuracy of our results. In addition, the Einstein 3D printer is recommended by Desktop Health to be used with FSU^+^ material which couldn’t be applicable in our study. This is considered another limitation.

Further studies are recommended to evaluate the effect of using different printers (including the Einstein 3D printer), curing protocols and printing layer thicknesses on the mechanical properties of the 3D printed hybrid resin material.

## Conclusions

Within the limitations of the current in-vitro study revealed that different printing angles (orientations) strongly affected the degree of conversion of 3D printed objects. The different groups showed variable degrees of surface roughness. The flexural strength and modulus of the 3D printed FSU^+^ material were affected by the different printing angles (orientations) being lower than those of LU and VE blocks. Specimens printed with a 45-degree angle showed the best results when compared to those printed with 0-and 90-degree angles. The fracture surfaces of the different groups revealed common features, indicating bending and showing different crack pathways.

## Data Availability

All data generated or analyzed during this study are included in this manuscript.

## Abbreviations

CAD/CAM: Computer-Aided Design / Computer-Aided Manufacturing
AM: Additive Manufacturing
3D: Three-Dimensional
DLP: Digital Light Processing
SLA: Stereolithography
VE: Vita Enamic
UDMA: Urethane Di-methacrylate
TEGDMA: Tri-ethylene Glycol Di-methacrylate
LU: Lava Ultimate
Bis-GMA: Bisphenol A Di-glycidyl Methacrylate
Bis-EMA: Bisphenol A Di-glycidyl Methacrylate Ethoxylated
FSU^+^: Flexcera Smile Ultra plus
FTIR: Fourier Transform Infrared Spectrophotometer
DC: Degree of Conversion
FS: Flexural Strength
FM: Flexural Modulus
SEM: Scanning Electron Microscope
LFED: Large Field Electron Detector
BSED: Back Scattered Electron Detector
SD: Standard Deviation
PICN: Polymer-infiltrated Ceramic-network
